# Reply to ‘Bias in energy system models with uniform cost of capital assumption’

**DOI:** 10.1038/s41467-019-12469-y

**Published:** 2019-10-09

**Authors:** Dmitrii Bogdanov, Michael Child, Christian Breyer

**Affiliations:** LUT University, Yliopistonkatu 34, 53850 Lappeenranta, Finland

**Keywords:** Environmental social sciences, Energy and society

**Replying to** Tobias Schmidt *Nature Communications* https://doi.org/10.1038/s41467-019-12468-z  (2019)

The commentary ‘Bias in energy system models with uniform cost of capital assumption’^1^ highlights the impact of the Cost of Capital (CoC) on the levelized cost of electricity (LCOE), and questions the choice of a uniform weighted average cost of capital (WACC) for all regions of the world. Since it is well known that CoC has significant impact on LCOE, having an accurate assumption is important for energy systems modeling. The commentary raises the relevant point that a uniform assumption could result in both underestimation and overestimation of the cost of renewable energy in different regions of the world. However, we consider it the best choice available for the transition period 2015 to 2050, as it was implemented in our study^2^. In addition, it is consistent with values published in leading scientific journals and international reports. To this end, we offer further explanation of the assumption used and discuss how modeling can be improved. The most relevant aspect of energy system scenario development is ignored in the commentary: modeling needs more accurate methods to project WACC/CoC for future periods up to 2050 and 2100 for all countries globally.

First, the commentary by Egli et al. would be applicable to any scientific publication or major international report in the field of energy system modeling. Currently, all major global energy system studies use uniform WACC assumptions, or quasi-uniform assumptions, as in the latest reports of the International Renewable Energy Association (IRENA). Our assumptions are in line with major scenarios: the International Energy Agency (IEA) uses 7% WACC and IRENA uses values of 7.5–10% in their latest reports. And the same WACC is uniform for all years in their scenarios. We use a WACC of 7%. Further, leading research teams in the world examining high shares of renewable energy for the global energy system analysis, such as Jacobson et al.^3^, Teske et al./German Aerospace Center^4^, DIW^5^, VTT^6^, and our team^2^, use similar uniform WACC assumptions.

Second, the commentary by Egli et al. shows that a challenge remains for the field of energy system analysis. The question of how to project WACC to 2050 and beyond remains unanswered in a satisfactory manner. Likewise, it must be acknowledged that regional differences of the future are difficult to predict and may not accurately be seen through a modern lens. There are many unknown factors of the future that could influence CoC in different regions. The same holds true for Integrated Assessment Models (IAMs), which are the basis of IPCC reports, as recently summarized for that specific community by Krey et al.^7^ for capital expenditures. This publication does not comment on variable WACC but assumes a uniform discount rate of 5%. The IAM scenarios are also heavily dependent on the choice of WACC assumptions due to typically wide use of nuclear energy and fossil carbon capture and storage technologies, which require substantial capital.

Third, while CoC values are very uncertain, current values for countries shown in the commentary are the result of an estimation method based on 10-year average German CoC values for PV projects and current Moody’s sovereign ratings^8^ and these values should be used for 2050 calculations. This is a questionable method of projecting future CoC and does nothing to improve upon the dominant method of using uniform values. A uniformly acceptable and better method for long-term WACC estimation for countries does not exist, which is not adequately reflected in the commentary, nor was a better method offered. Analysis of ratings history indicates that many countries have shown significant changes in risk levels in the past decades. South Korea as an example is not unique as many Eastern European countries show substantial risk reductions. At the same time, the countries with the highest CoC, as shown in the commentary, are unstable countries experiencing civil unrest. However, it would be strange to discuss a transition towards 100% RE in these countries while considering that current civil disorders continue until 2050 or 2100. So, it would be inaccurate to use a current CoC value for the long term.

Nevertheless, WACC has the highest impact on capital intensive technologies, like renewable energy generation and storage technologies, while conventional power generation is not that strongly affected. This makes it most vital to have realistic WACC assumptions in the steps leading towards the end of a transition, closer to the year 2050, when the share of RE in the energy system becomes dominant. In essence, our WACC assumption is for the year 2050, and then this value is also used for previous years. Since future CoC estimation methods for individual countries are not available, and direct extrapolation of current rates for all countries would be oversimplification, we use the uniform CoC of 7%, which is in line with other studies and is the best available assumption for 2050 so far.

Country specific CoC values could be used for early transition years but this begs the question of how the current CoC would converge toward the 2050 assumption. Therefore, we would appreciate support from our colleagues to design a more accurate method of estimation. In the end, this may not have decisive impact on the transition but would improve energy system scenarios. Another influential element to consider would be international development banks, which finance many energy projects in developing countries with lower interest rates than are available from local financing. This must also be considered to calculate the actual CoC values for global and regional energy systems. In the commentary it was suggested to use the country specific CoC of 2017 for all years including 2050, which we reject as a better solution to the uniform values chosen.

We strongly appreciate that the commentary introduces a core need in this field of study: an accurate method to project WACC from the present to future, in particular for the period of 2050 to 2100. This is what truly is needed for advancement of the state-of-the-art for sustainable energy transitions and achieving the Paris Agreement or United Nations Sustainable Development Goals. We support the critique of present CoC values, but this is of no practical help as the main question remains of how to project WACC (or CoC) to 2050 and beyond.

It is unquestionable that a proper choice of CoC assumption has a strong impact on the results of any energy system analysis and its uncertainty has to be considered in an analysis of results, which is mentioned in our publication. However, the approach suggested in the commentary with synthetic current CoC used for all years can result in greater distortion of the results. The case of Sudan highlighted in the commentary is a good example of such distortion. The country recently endured a civil war which resulted in a very high CoC. However, it appears highly unlikely that such a value will still be applicable in 2050. Current CoC should not be applied to 2050 systems as many conditions may change regionally in the next 30 years. This, too, will result in both underestimation and overestimation in several regions of the world. Since there is still no better method on offer to estimate future CoC values, uniform CoC remains the best solution available.

## Data Availability

The data that support the findings of this study are available from the authors on reasonable request.
